# Colchicine for cardiovascular and limb risk reduction in Medicare beneficiaries with peripheral artery disease: emulation of target trials

**DOI:** 10.1093/ehjopen/oeae062

**Published:** 2024-08-13

**Authors:** Patrick Heindel, James J Fitzgibbon, Eric Secemsky, Deepak L Bhatt, Mohammed Al-Omran, Subodh Verma, Ibrahim A Almaghlouth, Arin Madenci, Mohamad A Hussain

**Affiliations:** Harvard Medical School, 25 Shattuck St, Boston, MA 02115, USA; Division of Vascular and Endovascular Surgery, Department of Surgery, Brigham and Women’s Hospital, 75 Francis Street, Boston, MA 02115, USA; Department of Surgery, Center for Surgery and Public Health, Brigham and Women’s Hospital, Boston, MA 02115, USA; Harvard Medical School, 25 Shattuck St, Boston, MA 02115, USA; Division of Vascular and Endovascular Surgery, Department of Surgery, Brigham and Women’s Hospital, 75 Francis Street, Boston, MA 02115, USA; Department of Surgery, Center for Surgery and Public Health, Brigham and Women’s Hospital, Boston, MA 02115, USA; Harvard Medical School, 25 Shattuck St, Boston, MA 02115, USA; Division of Cardiovascular Medicine, Department of Medicine, Beth Israel Deaconess Medical Center, Boston, MA 02115, USA; Richard A. and Susan F. Smith Center for Outcomes Research, Beth Israel Deaconess Medical Center, Boston, MA 02115, USA; Mount Sinai Heart, Icahn School of Medicine at Mount Sinai Health System, 1 Gustave L Levy Pl, New York, NY 10029, USA; Division of Vascular Surgery and Li Ka Shing Knowledge Institute, St Michael’s Hospital, University of Toronto, 30 Bond St, Toronto, ON, M5B 1W8, Canada; Department of Surgery, King Faisal Specialist Hospital and Research Center, 7626 Al Takhassusi Al Far'i - Al Mathar District, Riyadh 12713 - 2613, Saudi Arabia; Division of Cardiac Surgery, St Michael’s Hospital, University of Toronto, 30 Bond St, Toronto, ON, M5B 1W8 Canada; Department of Medicine, Rheumatology Unit, King Saud University College of Medicine, RGSA3093, Riyadh, 12372 - 7065, Saudi Arabia; Harvard Medical School, 25 Shattuck St, Boston, MA 02115, USA; Department of Surgery, Boston Children’s Hospital, 300 Longwood Ave, Boston, MA 02115, USA; CAUSALab, Harvard T.H. Chan School of Public Health, 677 Huntington Ave, Boston, MA 02115, USA; Harvard Medical School, 25 Shattuck St, Boston, MA 02115, USA; Division of Vascular and Endovascular Surgery, Department of Surgery, Brigham and Women’s Hospital, 75 Francis Street, Boston, MA 02115, USA; Department of Surgery, Center for Surgery and Public Health, Brigham and Women’s Hospital, Boston, MA 02115, USA

**Keywords:** Colchicine, Inflammation, Peripheral artery disease, Vascular medicine, Comparative effectiveness

## Abstract

**Aims:**

Recent evidence from randomized trials demonstrates that colchicine can reduce the risk of major adverse cardiovascular events (MACE) in patients with coronary artery disease. Colchicine’s effect on lower-extremity peripheral artery disease (PAD) is not known.

**Methods and results:**

To make inferences about the real-world effectiveness of colchicine in PAD, we emulated two target trials leveraging the variable prescribing practice of adding colchicine vs. a non-steroidal anti-inflammatory drug (NSAID) to urate-lowering therapy in patients with gout and PAD. Emulated Trial 1 compared colchicine initiators with NSAID initiators. Emulated Trial 2 compared long-term (indefinite) and short-term (3 months) treatment strategies after initiating colchicine. Eligible individuals were those continuously enrolled in Medicare receiving care at a multicentre academic health system between July 2007 and December 2019. The primary outcome for both trials was a 2 year composite of major adverse limb events (MALE), MACE, and all-cause mortality. Secondary outcomes included MALE and death, MACE and death, and individual components of the primary outcome. Inverse probability weighting was used to adjust for confounding. Percentile-based 95% confidence intervals (CIs) were estimated using non-parametric bootstrapping. A total of 1820 eligible patients were included; the mean age was 77 years [standard deviation (SD) 7], 32% were female, and 9% were non-White. The mean (SD) duration of colchicine and NSAID therapy was 247 (345) and 137 (237) days, respectively. In the emulation of Trial 1, the risk of the primary composite outcome of MALE, MACE, and death at 2 years was 29.9% (95% CI 27.2%, 32.3%) in the colchicine group and 31.5% (28.3%, 34.6%) in the NSAID group, with a risk difference of −1.7% (95% CI −6.5%, 3.1%) and a risk ratio of 0.95 (95% CI 0.83, 1.07). Similar findings were noted in the emulation of Trial 2, with a risk of the primary composite outcome at 2 years of 30.7% (95% CI 23.7%, 38.1%) in the long-term colchicine group and 33.4% (95% CI 29.4%, 37.7%) in the short-term group, with a risk difference of −2.7% (95% CI −10.3%, 5.4%) and risk ratio of 0.92 (95% CI 0.70, 1.16).

**Conclusion:**

In a real-world sample of patients with PAD and gout, estimates of the effect of colchicine were consistent across two analyses and provided no conclusive evidence that colchicine decreased the risk of adverse cardiovascular or limb events and death. The cardiovascular and limb benefits of colchicine in older, comorbid populations with PAD and advanced systematic atherosclerosis remain uncertain.

**Editorial for this article:**  ***Eur Heart J Open***  **2024;4:oeae063, https://doi.org/10.1093/ehjopen/oeae063**

## Introduction

Prior translational research has established that the underlying pathophysiology of atherosclerosis is characterized by chronic inflammation of the arterial wall through intense immunological activity.^1,2^ Clinical evidence has linked the cardiovascular benefits of statins not only to their lipid-lowering properties, but also to their systemic anti-inflammatory effects.^3–12^ Recently, trials have provided evidence that the anti-inflammatory drug colchicine, most often used in the treatment and prevention of gout flares, has efficacy in reducing major adverse cardiovascular events (MACE) in patients with atherosclerotic coronary artery disease.^10,11,13–16^ Colchicine’s potential for reducing MACE and major adverse limb events (MALE) among individuals with lower-extremity peripheral artery disease (PAD) remains unknown. A randomized trial to assess the effect of colchicine in patients with PAD is currently enrolling and should provide evidence to better understand which populations may benefit from secondary prevention of MACE and MALE with colchicine. Trial results will not be available for years, and well-designed observational comparative effectiveness studies are the best available evidence for clinicians making decisions today.

Peripheral artery disease affects over 200 million people globally, and it remains underdiagnosed and undertreated.^17^ Despite advancements in secondary prevention therapy over the last two decades, the 5 year mortality risk in patients suffering from advanced PAD has remained high at 50%.^18,19^ More research is urgently needed to establish therapies that can positively affect the prognosis of individuals with PAD and improve cardiovascular and limb-related outcomes. Given the demonstrated efficacy of colchicine in chronic coronary disease, its investigation as a potential therapy for PAD holds immense promise.

The present study is an observational cohort study designed to emulate hypothetical randomized trials (‘target trials’) that would be performed to study the effectiveness of treatment with colchicine on the prevention of adverse cardiovascular and limb events. By leveraging the variable prescribing practice of adding colchicine vs. a non-steroidal anti-inflammatory drug (NSAID) to urate-lowering therapy in patients with gout, it may be possible to make inferences about the effects of colchicine in PAD using observational data. The hypothesis driving the present study was that in patients with comorbid gout and PAD, initiation of urate-lowering therapy with low-dose colchicine (vs. NSAID) would be associated with a decreased risk of major adverse cardiovascular and limb events at 2 years. Additionally, we hypothesized that the effect of colchicine would be related to the duration of therapy, with longer-term colchicine use resulting in additional protection from adverse cardiovascular and limb events when compared with shorter-term use.

## Methods

In this section, a description of how two hypothetical target trials would be designed and analysed is provided: the first comparing colchicine with NSAID treatment and the second comparing a shorter with a longer duration of colchicine treatment. Each aspect of the design and analysis of these target trials is then explicitly emulated using observational data.^20–22^ The protocol for the target trials and their emulation is summarized in Supplementary material online, *Table S1* (Trial 1) and *Table S2* (Trial 2). The present work was approved by the Mass General Brigham (MGB) Human Research Committee Institutional Review Board for the use of electronic health records and insurance claims data in research.

### Specification of the target trials

Eligibility criteria would be the same for both target trials and would include patients enrolled in Medicare Parts A, B, and D receiving care at any MGB facility between 1 July 2007 and 31 December 2019; use of the MGB health system in the 6 months prior to enrolment; age >66 years at time of trial enrolment; diagnoses of both PAD and gout; initiation of urate-lowering therapy; and no recent use of NSAIDs, colchicine, or urate-lowering therapy in the prior 6 months. Urate-lowering therapy would include the drugs allopurinol and febuxostat. Additionally, patients with contraindications to either treatment strategy would not be eligible for trial participation, which includes patients with relevant drug allergies, hepatic failure, or advanced chronic kidney disease (CKD; Stage 4, 5, or end-stage kidney disease). The same eligibility criteria were applied to both target trials for comparability.

Individuals in Trial 1 would be randomized to the initiation of either colchicine (treatment) or an NSAID (control) as prophylaxis for gout flares when initiating urate-lowering therapy. The pragmatic trial would be non-blinded, and the duration and intensity of therapy would be guided by the treating clinician’s judgement. Prescription NSAIDs would include naproxen, indomethacin, ibuprofen, diclofenac, meloxicam, and celecoxib.

Individuals in Trial 2 would be randomized to the initiation of long-term colchicine therapy (treatment) or short-term colchicine therapy (control) when initiating urate-lowering therapy. In this trial, the protocol would dictate limits for the duration of therapy, with the long-term arm being randomized to take colchicine daily indefinitely, and the short-term arm taking colchicine daily for 3 months (90 days) and then stopping treatment.

The primary composite outcome for both trials would be any MACE, MALE, or all-cause mortality, assessed at 2 years of follow-up to align with prior randomized trials.^11,15^ A MACE was defined as hospital admission for acute myocardial infarction, acute stroke (including transient ischaemic attack), or coronary revascularization (open or percutaneous). A MALE was defined as above-ankle amputation, iliac arterial revascularization, or infrainguinal arterial revascularization (open or percutaneous). Secondary outcomes included the individual components of the primary outcome, a composite of MALE and death, and a composite of MACE and death.

The date at which each trial participant met all eligibility criteria and was randomized to a treatment arm would define the start of trial follow-up. Follow-up would continue until the occurrence of an outcome of interest, Medicare plan disenrolment, or the conclusion of the trial on 31 December 2019, whichever were to occur first.

In either trial, the intention-to-treat effect could be estimated (i.e. patients analysed according to the group to which they were assigned, regardless of their adherence to therapy) by comparing the proportion of individuals who developed the outcome of interest in each assignment arm. Alternatively, practicing clinicians may be more interested in the per-protocol effect (i.e. the effect had all patients adhered to their assigned protocol). To estimate the per-protocol effect, individuals would be analysed according to the arm they were observed to follow, and the analysis would be adjusted for baseline covariates prognostic of the outcome that also predict adherence to treatment assignment using stabilized inverse probability (IP) weighting.^23^ These covariates would include age at enrolment, sex, race, medical comorbidities (coronary artery disease, heart failure, cardiac arrythmia, history of MACE, CKD, chronic pulmonary disease, diabetes, hypertension), general health as quantified by the Elixhauser score,^24^ gout disease severity (number of prior gout attacks or encounters with a primary diagnosis of gout, use of steroids), PAD severity (rest pain, tissue loss, history of MALE), medication history (insulin, beta-blockers, prescription antiplatelets, anticoagulants, cilostazol, and number of antihypertensives), calendar time, and duration of plan enrolment prior to treatment initiation. Subgroup analyses would be performed in the group of patients with coronary artery disease present at baseline to more closely replicate the population enrolled in prior trials.^11^ Additional subgroup analyses would be performed after excluding patients with coronary artery disease to address the potential bias introduced by contraindications to NSAID therapy.

In Trial 2 (long- vs. short-term colchicine), in which the protocol specifies a sustained regime, a cloning, censoring, and weighting approach is helpful to estimate effects free from confounding and selection bias (i.e. collider stratification bias).^25,26^ To estimate the per-protocol effect of the duration of colchicine therapy, data from the same group of patients who were eligible for Trial 1 at baseline would be cloned (i.e. duplicated, so that two copies of identical data were present). Time would be discretized using month-long (30-day) intervals. Each clone would be assigned to both the long- and the short-term treatment arms. Then, each clone would be censored if and when their data were found to be incompatible with the assigned treatment protocol (i.e. at the time of protocol deviation). Finally, IP weights would be estimated using propensity scores for remaining uncensored at each time point. The covariates for the censoring propensity scores would be the same as those used in Trial 1, with the addition of steroid use as a time-varying covariate as a proxy for gout flares. A pooled logistic regression outcome model would then be fit using the weights generated in the prior step. Linear, quadratic, and cubic terms would be used for time, as well as an interaction term between time and the treatment arm.

The risk of each outcome would be estimated using the Kaplan–Meier method for Trial 1 and weighted pooled logistic outcome regression for Trial 2. Percentile-based confidence intervals (CIs) at the 95% level would be generated for all outcome estimates using non-parametric bootstrapping and 500 resamples. Inverse probability weights would be truncated at the 99th percentile. Mean differences (standardized mean differences [SMD] for continuous variables) in relevant covariates would be calculated before and after weighting. Mean differences exceeding 10% would be considered indicative of residual imbalances.^27^ Descriptive statistics would be presented as a count (percentage) and a mean [standard deviation (SD)]. In the analyses of MALE and MACE alone, the competing risk of mortality would be treated as a non-censoring event to estimate the total effect.^28^ When describing the specific events resulting in MACE or MALE, if patients experienced multiple events on the same day, the most invasive intervention would be reported (i.e. if amputation and angioplasty occurred on the same date, amputation would be reported as the MALE). The number-needed to treat (NNT) and the number-needed to harm (NNH) would be calculated as the inverse of the absolute risk reduction.

### Emulation of the target trials

The trials described above were emulated using data from the MGB electronic health record data warehouse linked to Medicare claims and pharmacy data from the Center for Medicare and Medicaid Services (CMS). The linkage process has been described in detail previously.^29^ The resulting linked data set provides granular demographic and clinical information for the determination of study eligibility and control for bias, along with health-system agnostic longitudinal follow-up for outcome ascertainment and protocol deviation assessment.

Peripheral artery disease and gout diagnoses were established by clinical encounters with a relevant International Classification of Diseases (ICD) version 9 or 10 diagnosis code in the primary position. Peripheral artery disease was additionally identified by either an ankle-brachial index <0.9 recorded in the MGB electronic health record or any prior revascularization or amputation for PAD. Medical histories were built using the ICD codes from the 6 months prior to candidate enrolment. The transition from ICD-9 to ICD-10, which occurred in 2015, was addressed using the General Equivalence Mappings developed by the National Center for Health Statistics at CMS.^30^

Medication use was identified with Medicare pharmacy dispensing claims. The duration of therapy was defined by the prescribed duration in the claim (i.e. the intended treatment).

Outcomes other than death were assessed using principal discharge diagnoses for inpatient stays defined by ICD codes or procedural interventions defined by either ICD procedure codes or Current Procedural Terminology codes. Cardiovascular-specific mortality is challenging to assess in observational data, and therefore, all-cause mortality was used due to the high degree of ascertainment in CMS data. Study variable definition details are listed in Supplementary material online, *Table S3*.

For the emulation of randomization at baseline, a pseudo-population was generated using IP weighting to adjust for covariates potentially associated with both the treatment assignment and the risk of the outcome. The covariates used for IP weighting were identical to those described in the section above. A logistic model was used to predict the probability of treatment, and the stabilized IP of treatment weights was calculated. Weighting was chosen over other methods (e.g. matching) to maximize statistical efficiency. All other aspects of the analyses were identical to those that would be performed in the target trials specified above. Analyses were performed using R version 4.2.1 and the packages *arrow*, *tidyverse*, and *survival*.^31–34^

## Results

### Description of the cohort

**Figure 1:**
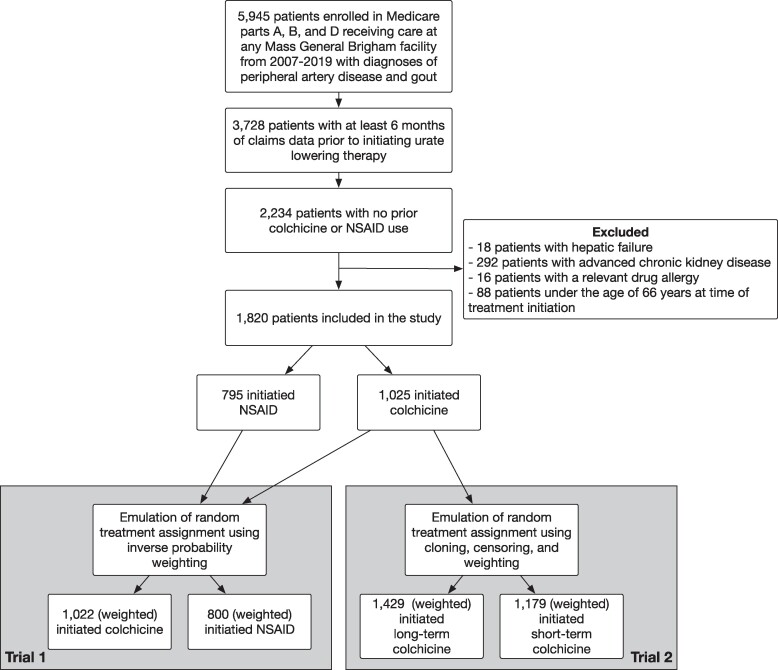
Selection of participants for the emulation of target trials evaluating the comparative effectiveness of colchicine vs. non-steroidal anti-inflammatory drugs (Emulated Trial 1) and long-term vs. short-term colchicine (Emulated Trial 2).

A total of 1820 patients were selected for study participation (*Figure 1*). All patients participated in Emulated Trial 1, with 1025 patients in the colchicine arm and 795 patients in the NSAID arm. The mean (SD) age was 77 (6.8) years, 31.7% were female, and 9.2% were non-white. The disease severity of PAD appeared low, with only 1.1% with documented rest pain, 11.9% of patients with tissue loss, and 1.9% having experienced a MALE in the past 6 months. The distribution of medical comorbidities was as expected for a cohort with PAD, reflecting a relatively high prevalence of hypertension, diabetes, and other manifestations of atherosclerosis. Notably, only 76.1% of the cohort was taking statins prior to study enrolment.

Regarding the specific drugs used for the treatment of gout, allopurinol was the most dispensed urate-lowering therapy by far (97.0%). The specific NSAIDs prescribed included diclofenac (*n* = 207, 26.0%), indomethacin (*n* = 203, 25.5%), ibuprofen (*n* = 163, 20.5%), naproxen (*n* = 112, 14.1%), celecoxib (*n* = 69, 8.7%), and meloxicam (*n* = 41, 5.2%). The mean (SD) duration of colchicine and NSAID therapy was 247 (345) and 137 (237) days, respectively.

*Table 1* describes the baseline characteristics of study participants before and after weighting. Imbalances in relevant covariates prior to weighting were minimal, with only atrial fibrillation, heart failure, anticoagulation use, Medicare enrolment duration, and the Elixhauser score exceeding the 10% SMD threshold prior to weighting. After weighting, no substantial residual imbalances were noted between the colchicine and the NSAID groups (see Supplementary material online, *Figure S1*).

**Table 1:** Baseline characteristics of participants

Characteristic	Unadjusted			Weighted		
	Colchicine (*n* = 1025)	NSAID (*n* = 795)	SMD	Colchicine (*n* = 1022)	NSAID (*n* = 800)	SMD
Age (years)	77.3 (6.9)	76.7 (6.8)	0.08	77.0 (6.8)	77.0 (6.9)	<0.01
Sex (male)	706 (68.9%)	537 (67.5%)	0.01	699 (68.4%)	550 (68.7%)	<0.01
Race (non-white)	91 (8.9%)	76 (9.6%)	0.01	94 (9.2%)	73 (9.2%)	<0.01
Obesity	188 (18.3%)	142 (17.9%)	<0.01	187 (18.3%)	149 (18.6%)	<0.01
Coronary artery disease	586 (57.2%)	403 (50.7%)	0.06	556 (54.4%)	433 (54.2%)	<0.01
Cerebrovascular disease	138 (13.5%)	76 (9.6%)	0.04	129 (12.6%)	85 (10.6%)	0.02
Heart failure	378 (36.9%)	214 (26.9%)	0.10	335 (32.8%)	264 (33.0%)	<0.01
Atrial fibrillation	523 (51.0%)	284 (35.7%)	0.15	455 (44.5%)	358 (44.8%)	<0.01
CKD (Stages 1–3)	181 (17.7%)	117 (14.7%)	0.03	168 (16.5%)	132 (16.5%)	<0.01
COPD	240 (23.4%)	168 (21.1%)	0.02	228 (22.3%)	176 (22.0%)	<0.01
Diabetes	529 (51.6%)	420 (52.8%)	0.01	531 (52.0%)	419 (52.4%)	<0.01
Insulin	154 (15.0%)	108 (13.6%)	0.01	148 (14.4%)	115 (14.4%)	<0.01
Antihypertensives (no.)						
0	197 (19.2%)	145 (18.2%)	0.01	194 (19.0%)	154 (19.2%)	<0.01
1	429 (41.9%)	309 (38.9%)	0.03	417 (40.8%)	326 (40.8%)	<0.01
2	281 (27.4%)	248 (31.2%)	0.04	293 (28.6%)	229 (28.7%)	<0.01
3+	118 (11.5%)	93 (11.7%)	<0.01	118 (11.6%)	91 (11.3%)	<0.01
Beta–blocker	758 (74.0%)	548 (68.9%)	0.05	735 (71.9%)	576 (72.1%)	<0.01
Statin	798 (77.9%)	587 (73.8%)	0.04	784 (76.7%)	615 (76.8%)	<0.01
Antiplatelet (prescription only)	154 (15.0%)	108 (13.6%)	0.01	148 (14.5%)	116 (14.5%)	<0.01
Cilostazol	25 (2.4%)	19 (2.4%)	<0.01	25 (2.4%)	20 (2.5%)	<0.01
Anticoagulation	341 (33.3%)	162 (20.4%)	0.13	286 (28.0%)	230 (28.7%)	0.01
Prior MALE	15 (1.5%)	14 (1.8%)	<0.01	17 (1.7%)	13 (1.6%)	<0.01
Prior MACE	66 (6.4%)	36 (4.5%)	0.02	58 (5.6%)	45 (5.6%)	<0.01
Rest pain	13 (1.3%)	7 (0.9%)	<0.01	12 (1.1%)	9 (1.2%)	<0.01
Tissue loss	141 (13.8%)	76 (9.6%)	0.04	123 (12.1%)	99 (12.4%)	<0.01
Elixhauser score	5.2 (2.9)	4.5 (2.8)	0.22	4.9 (2.8)	4.9 (3.0)	<0.01
Calendar time^a^	83 (41)	85 (41)	0.03	84 (41)	84 (41)	<0.01
Enrolment time^b^	58 (37)	62 (38)	0.11	60 (38)	60 (37)	<0.01
Prior gout encounters	111 (10.8%)	53 (6.7%)	0.04	92 (9.0%)	73 (9.2%)	<0.01
Febuxostat (vs. allopurinol)	32 (3.1%)	22 (2.8%)	<0.01	30 (2.9%)	23 (2.9%)	<0.01
Prior steroid use	444 (43.3%)	270 (34.0%)	0.09	404 (39.5%)	317 (39.6%)	<0.01

Both unadjusted totals and totals in the pseudo-population created by IP weighting are reported.

ASA, acetylsalicylic acid, aspirin; CKD, chronic kidney disease; COPD, chronic obstructive pulmonary disease; MACE, major adverse cardiovascular events; MALE, major adverse limb events; NSAID, non-steroidal anti-inflammatory drug; SMD, standardized mean difference (continuous variables) or mean difference (categorical variables).

^a^Measured in number of 30 day blocks since study start (July 2007).

^b^Measured in number of 30 day blocks of Medicare enrolment prior to study inclusion.

### Emulated Trial 1 results

**Figure 2:**
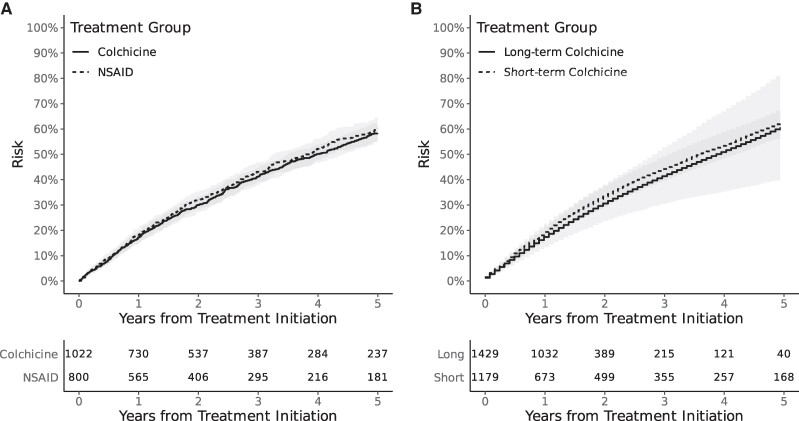
Risk of the primary composite outcome of major adverse limb events, major adverse cardiovascular events, or death for Emulated Trial 1 (*A*) and Emulated Trial 2 (*B*) from treatment initiation to 5 years. The solid line represents the treatment group [colchicine (*A*) or long-term colchicine (*B*)], and the dotted line represents the control [NSAID (*A*) or short-term colchicine (*B*)]. The shaded area represents percentile-based 95% confidence intervals generated with bootstrapping. The weighted number of participants at risk for the outcome (cloned in Emulated Trial 2) is included in the corresponding table. MACE, major adverse cardiovascular events (acute myocardial infarction, stroke, coronary revascularization); MALE, major adverse limb events (above-ankle amputation, bypass graft, stenting, graft revision, thrombolysis, thrombectomy); NSAID, non-steroidal anti-inflammatory drug.

In the emulated trial comparing the initiators of colchicine with the initiators of NSAIDs for gout flare prophylaxis while starting urate-lowering therapy, the intention-to-treat risk of the primary composite outcome of MALE, MACE, or death at 2 years was 29.9% (95% CI 27.2, 32.3) in the colchicine group and 31.5% (95% CI 28.3, 34.6) in the NSAID group (*Figure 2A*). The risk difference at 2 years was −1.7% (95% CI −6.5, 3.1), which corresponds to an NNT of 59 and a CI compatible with an NNT of 15 and NNH of 32. The risk ratio at 2 years was 0.95 (95% CI 0.83, 1.07).

Among patients who experienced a MALE, 28.6% had a below-knee amputation, 20.4% had an infrainguinal stent, 11.7% underwent infrainguinal bypass, 5.8% had an above-knee amputation, and 5.3% underwent thromboendarterectomy, with the remaining events occurring in fewer than 5% of patients. Among patients who experienced a MACE, 35.9% were admitted with a new primary discharge diagnosis of acute myocardial infarction, 25.8% were admitted for an acute stroke or transient ischaemic attack, 25.0% underwent percutaneous coronary intervention, and 13.3% underwent a coronary artery bypass.

**Figure 3:**
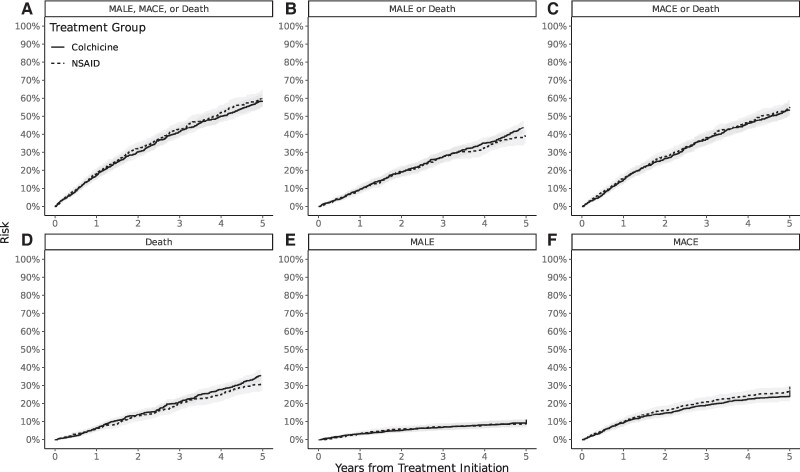
Risk of the primary and secondary outcomes in Emulated Trial 1 from treatment initiation to 5 years. Panels plot the risk of the outcomes MACE, MALE, or Death (A), MALE or Death (B), MACE or Death (C), Death (D), MALE (E), MACE (F). The solid line represents the treatment group (colchicine), and the dotted line represents the control (non-steroidal anti-inflammatory drug). The shaded area represents percentile-based 95% confidence intervals generated with bootstrapping. MACE, major adverse cardiovascular events (acute myocardial infarction, stroke, coronary revascularization); MALE, major adverse limb events (above-ankle amputation, bypass graft, stenting, graft revision, thrombolysis, thrombectomy); NSAID, non-steroidal anti-inflammatory drug.

All estimates for secondary outcomes were most consistent with small effect sizes with CIs compatible with both increased and decreased risk (*Figure 3*). Unadjusted and weighted estimates for all outcomes at 2 years of follow-up are reported in *Table 2*.

**Table 2:** Trial 1 effect estimates at 2 years of follow-up

Outcome	Estimate	Unadjusted	Weighted
MALE, MACE, or death	Risk (colchicine)	31.7% (29.0, 34.5)	29.9% (27.2, 32.3)
	Risk (NSAID)	30.7% (28.1, 33.5)	31.5% (28.3, 34.6)
	Risk difference	1.3% (−3.4, 6.0)	−1.7% (−6.5, 3.1)
	Risk ratio	1.03 (0.90, 1.17)	0.95 (0.83, 1.07)
MALE or death	Risk (colchicine)	19.9% (17.9, 22.1)	18.2% (16.2, 20.3)
	Risk (NSAID)	17.4% (15.0, 19.6)	19.0% (16.2, 21.7)
	Risk difference	2.5% (−1.4, 6.4)	−0.7% (−4.7, 3.3)
	Risk ratio	1.14 (0.94, 1.37)	0.95 (0.80, 1.15)
MACE or death	Risk (colchicine)	28.8% (26.3, 31.1)	26.9% (24.5, 29.4)
	Risk (NSAID)	27.0% (24.3, 29.5)	27.7% (24.9, 30.7)
	Risk difference	1.8% (−2.5, 6.2)	−0.7% (−5.2, 3.8)
	Risk ratio	1.06 (0.94, 1.21)	0.97 (0.84, 1.11)
Death	Risk (Colchicine)	15.2% (13.1, 17.5)	13.7% (11.9, 15.5)
	Risk (NSAID)	12.3% (10.3, 14.5)	13.3% (11.2, 15.7)
	Risk difference	3.1% (−0.6, 6.8)	0.6% (−2.8, 4.0)
	Risk ratio	1.23 (0.99, 1.57)	1.03 (0.83, 1.26)
MALE	Risk (colchicine)	4.5% (3.5, 5.6)	4.5% (3.6, 5.5)
	Risk (NSAID)	4.8% (3.6, 6.2)	5.7% (4.2, 7.5)
	Risk difference	−0.2% (−2.2, 1.8)	−1.1% (−3.3, 1.2)
	Risk ratio	0.94 (0.65, 1.35)	0.80 (0.57, 1.14)
MACE	Risk (colchicine)	15.9% (14.2, 17.8)	15.3% (13.4, 17.1)
	Risk (NSAID)	15.8% (13.6, 17.9)	16.4% (14.3, 18.8)
	Risk difference	−0.1% (−3.5, 3.3)	−1.1% (−4.5, 2.4)
	Risk ratio	0.99 (0.85, 1.20)	0.93 (0.78, 1.13)

The crude unweighted estimates are presented along with the IP-weighted estimates. Risks and risk differences are reported as percentages. 95% CIs generated with bootstrapping are reported with all estimates.

MACE, major adverse cardiovascular events (admission for myocardial infarction, acute stroke, or coronary revascularization); MALE, major adverse limb events (above-ankle amputation, iliac or infrainguinal arterial revascularization); NSAID, non-steroidal anti-inflammatory drug.

### Emulated Trial 2 results

Emulated Trial 2 compared two sustained treatment strategies of either long-term colchicine or short-term colchicine. The per-protocol risk of the primary composite outcome of MALE, MACE, or death at 2 years was 30.7% (95% CI 23.7, 38.1) in the long-term colchicine group and 33.4% (95% CI 29.7, 37.7) in the short-term colchicine group (*Figure 2B*). The risk difference at 2 years was −2.7% (95% CI −10.3, 5.4), which corresponds to an NNT of 37 and a CI compatible with an NNT of 10 and NNH of 19. The risk ratio at 2 years was 0.92 (95% CI 0.70, 1.16). These results are largely compatible with the results of Emulated Trial 1.

**Figure 4:**
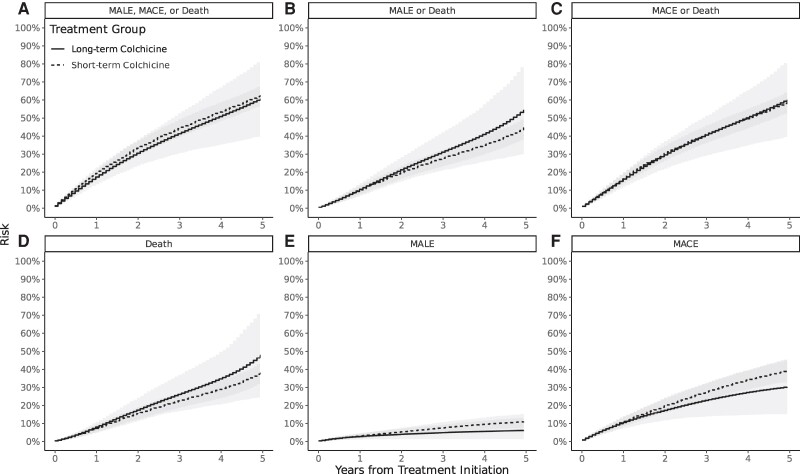
Risk of the primary and secondary outcomes in Emulated Trial 2 from treatment initiation to 5 years. Panels plot the risk of the outcomes MACE, MALE, or Death (A), MALE or Death (B), MACE or Death (C), Death (D), MALE (E), MACE (F). The solid line represents the treatment group (long-term colchicine), and the dotted line represents the control (short-term colchicine). The shaded area represents percentile-based 95% CIs generated with bootstrapping. MACE, major adverse cardiovascular events (acute myocardial infarction, stroke, coronary revascularization); MALE, major adverse limb events (above-ankle amputation, bypass graft, stenting, graft revision, thrombolysis, thrombectomy); NSAID, non-steroidal anti-inflammatory drug.

Estimates for secondary outcomes in Emulated Trial 2 were similar to Emulated Trial 1 with wider CIs. Notable secondary outcomes in Emulated Trial 2 included weak evidence for a decrease in both the risk of MALE and MACE alone at 2 years, but a corresponding small increase in the risk of all-cause mortality (*Figure 4*). Beyond the primary time point of 2 years, point estimates of risks in the long-term group diverged from the short-term group, but CIs also widened (*Figure 4*). Unadjusted and weighted estimates for all outcomes at 2 years of follow-up are reported in *Table 3*. Pointwise risk differences are plotted in Supplementary material online, *Figure S2*.

**Table 3:** Trial 2 effect estimates at 2 years of follow-up

Outcome	Estimate	Weighted
MALE, MACE, or death	Risk (long term)	30.7% (23.7, 38.1)
	Risk (short term)	33.4% (29.4, 37.7)
	Risk difference	−2.7% (−10.3, 5.4)
	Risk ratio	0.92 (0.70, 1.16)
MALE or death	Risk (long term)	21.5% (15.2, 28.5)
	Risk (short term)	19.9% (16.7, 23.6)
	Risk difference	1.5% (−5.6, 8.9)
	Risk ratio	1.09 (0.73, 1.48)
MACE or death	Risk (long term)	29.7% (22.1, 37.4)
	Risk (short term)	30.5% (26.7, 35.0)
	Risk difference	−0.7% (−9.0, 7.3)
	Risk ratio	0.98 (0.72, 1.25)
Death	Risk (long term)	17.7% (11.5, 24.5)
	Risk (short term)	15.7% (12.5, 19.2)
	Risk difference	2.0% (−5.0, 8.7)
	Risk ratio	1.14 (0.72, 1.60)
MALE	Risk (long term)	4.1% (1.4, 7.3)
	Risk (short term)	5.4% (3.6, 7.8)
	Risk difference	−1.3% (−5.1, 2.0)
	Risk ratio	0.78 (0.23, 1.41)
MACE	Risk (long term)	17.6% (11.8, 24.2)
	Risk (short term)	19.9% (16.3, 24.6)
	Risk difference	−2.3% (−9.1, 5.0)
	Risk ratio	0.89 (0.58, 1.27)

Inverse probability–weighted estimates are presented. Risks and risk differences are reported as percentages. 95% CIs generated with bootstrapping are reported with all estimates.

MACE, major adverse cardiovascular events (admission for myocardial infarction, acute stroke, or coronary revascularization); MALE, major adverse limb events (above-ankle amputation, iliac, or infrainguinal arterial revascularization); NSAID, non-steroidal anti-inflammatory drug.

### Subgroups with and without prevalent coronary artery disease

All analyses were performed in the subgroup of patients with coronary artery disease present at baseline (*n* = 989) to more closely replicate the population enrolled in trials such as LoDoCo2.^11^ No effect modification in this subgroup was noted, with similar direction and magnitude of estimates for the primary and all secondary outcomes, although lack of precision limits interpretation. Analyses were additionally performed in the subgroup of patients without coronary artery disease (*n* = 831). Estimates for these subgroup analyses are presented in Supplementary material online, *Figures S3–S6*.

## Discussion

Using real-world data from a large multicentre academic healthcare system in the USA, we emulated two hypothetical target trials to estimate the effect of colchicine use on adverse cardiovascular events, adverse limb events, and mortality in a high-risk cohort of patients with comorbid gout and PAD.

This study was intended to extend the results of recent trials demonstrating a risk reduction effect of colchicine in patients with chronic coronary disease to patients with PAD. After emulating two target trials designed to answer related study questions, we were unable to demonstrate convincing evidence for a risk reducing effect of colchicine in our study cohort. Emulated Trial 1 compared the initiators of colchicine with a control group initiating NSAIDs when starting urate-lowering therapy, estimating the observational analogue of an intention-to-treat effect. Emulated Trial 2 compared short- and long-term colchicine use, estimating a per-protocol effect with methods that avoid immortal-time bias and adjust for time-varying confounding. The results of both emulated trials were concordant, estimating effects compatible with a small reduction or small increase in the risk of the primary composite outcome of MACE, MALE, and all-cause mortality at 2 years.

Atherosclerosis is known to be closely linked to systemic inflammation.^1,2^ Colchicine is thought to reduce MACE through a reduction of systemic inflammation.^10,35,36^ In the two largest contemporary trials investigating colchicine use in coronary disease, LoDoCo2 (Low-Dose Colchicine for Secondary Prevention of Cardiovascular Disease 2) and COLCOT (Colchicine Cardiovascular Outcomes Trial), the risk of the primary cardiovascular outcome overall (5.5–9.6%) was substantially lower than in the present study (29.9% colchicine, 31.5% NSAID at 2 years).^11,15^ LoDoCo2 enrolled 5522 patients with chronic coronary disease and randomized them to daily colchicine or placebo, reporting a hazard ratio of 0.69 (95% CI 0.57, 0.83) for the primary outcome of MACE (including cardiovascular death) at the median follow-up of 28.6 months.^11^ In contrast to the patients in our study, LoDoCo2 enrolled patients with a mean age of 66 years (vs. 77 years), of whom only 18.2% (vs. 52.1%) had diabetes and 5% (vs. 14.4%) were insulin dependent. Additionally, baseline medical therapy for the treatment and prevention of atherosclerosis was more optimal in the LoDoCo2 cohort, with 96.6% (vs. 76.1%) taking a lipid-lowering drug, 99.7% (vs. 14.4%, excluding over-the-counter aspirin) taking an antiplatelet, and 71.7% (vs. 63.7%) taking an inhibitor of the renin–angiotensin system. The patients enrolled in COLCOT exhibited similar patterns of comorbidities and baseline optimal medical therapy to LoDoCo2.^15^ These differences in study cohorts raise the possibility of effect modification by either atherosclerosis disease severity or concurrent optimal medical therapy as the reason for differences in effect estimates. Although this study did not include direct measures of atherosclerosis disease severity, PAD itself is typically associated with a more advanced systemic atherosclerotic burden than isolated coronary disease—perhaps this contributed to the diminished effect of colchicine in this study when compared with trials enrolling patients with coronary disease.^37–39^ Randomized trials, such as LEADER-PAD (ClinicalTrials.gov NCT04774159), have been developed and are currently enrolling to answer the question of whether colchicine is effective as a means of secondary prevention of atherosclerotic adverse events in patients with PAD.

Target trial emulation is a framework for the development of observational cohort studies that align with principles used in randomized trials. Emulating target trials helps prevent common sources of bias unintentionally introduced in the analysis of observational data, improves the transparency of observational comparative effectiveness research, and makes study goals explicit. In this study, some specific methodological choices warrant discussion.

For clinical decision-making and to prevent prevalent user bias, initiators of colchicine were most of interested. However, comparing initiators with non-initiators in observational data analysis is often fraught with intractable selection bias and confounding by indication—the patient starting colchicine is, by definition, different than a patient not starting colchicine. If the clinical differences between initiators and non-initiators are also associated with any of the outcomes of interest, effect estimates will be biased. Therefore, to conduct a study with an incident-user, active comparator design, the inciting clinical event prompting initiation of prophylaxis (colchicine or NSAID) is identical. In such a design, the potential for confounding is diminished, even without additional statistical adjustment.^40^

Another challenge in the analysis of observational data is the investigation of the effect of treatment duration. Comparing long-term with short-term users of any treatment will result in substantial immortal-time bias when using standard epidemiologic techniques.^21,26,41^ Intuitively, patients who adhere to a treatment for a long duration must survive a long duration. Comparing short- and long-term users naïvely will nearly always lead to the conclusion that long-term use is beneficial, simply because long-term users had to survive longer to take the treatment. The methods of cloning, censoring, and weighting employed in Emulated Trial 2 is one way to avoid introducing this important source of bias.^25^

Despite these strengths, there remain inherent limitations to the study. The present work was limited to patients with gout. If gout is an effect modifier in the relationship between colchicine and the outcome, the results of our study may not apply to the broader population of patients with PAD. Additionally, real-world colchicine prescribing patterns differ from those specified in the target trials, with a mean duration of treatment with colchicine of less than a year. As in any study making secondary use of health records and billing codes for ascertainment of medical diagnoses, there is likely some degree of misclassification of covariates. Another concern may be that NSAIDs are generally contraindicated in patients with coronary disease, and as such, enrolling these patients in a trial comparing NSAIDs with colchicine would be unethical; however, NSAIDs are still prescribed in real-world practice, enabling the design of Emulated Trial 1. Although NSAIDs would be relatively contraindicated for some of these patients, the treating providers who used NSAIDs either were not aware of these patients’ contraindications or made a clinical decision that the marginal benefit of the NSAID outweighed the marginal risk. Additionally, if the NSAIDs prescribed to patients in this study caused an increase in MACE, one may expect the estimates to be biased towards a beneficial effect of colchicine—we did not observe this. Fortunately, Emulated Trial 2 avoids any potential bias introduced by providers being hesitant to prescribe NSAIDs to patients with advanced cardiovascular disease by comparing short- and long-term colchicine users. Finally, residual confounding is impossible to rule out in any observational study, although the incident-user, active comparator design substantially reduces the potential for confounding bias.

Unfortunately, this study was unable to convincingly replicate the MACE reduction benefit of colchicine that has been demonstrated in recent trials of patients with chronic coronary disase.^15^ This is due at least in part to statistical limitations—the additional stratification by the documented presence of chronic coronary disease substantially reduced the size of the sample and limited the precision of our estimates. The estimates of colchicine’s effect on MACE in patients with coronary disease were consistent with harm, no effect, or reduction of MACE—these results do not contradict prior trials but are simply less precise.

## Conclusions

The estimates for the effect of colchicine were consistent across two cohort studies deigned to emulate hypothetical target trials and provided no conclusive evidence that colchicine reduced cardiovascular or limb events and death in a real-world sample of patients with PAD and gout.

## Supplementary Material

oeae062_Supplementary_Data

## Data Availability

De-identified data used for the analyses presented in this work are available to researchers on request with the corresponding author.

## Lead author biography

Patrick Heindel, MD, MPH, is a chief resident in general surgery at the Brigham and Women’s Hospital/Harvard Medical School. He received his Doctor of Medicine degree from the Keck School of Medicine of the University of Southern California and his Masters of Public Health from the Harvard T.H. Chan School of Public Health. He completed an NIH T32 research fellowship in the Harvard–Longwood Research Training in Vascular Surgery Program, as well as a fellowship at the Brigham and Women’s Hospital Center for Surgery and Public Health. Dr Heindel’s research interests include leveraging data science techniques and causal inference methods to provide better evidence-based care for patients suffering from vascular disease.
