# Supplementary material for: Colchicine for cardiovascular and limb risk reduction in Medicare beneficiaries with peripheral artery disease: emulation of target trials
Source: Eur Heart J Open. 2024 Aug 13;4(4):oeae062. doi: 10.1093/ehjopen/oeae062 (PMC11339712; doi:10.1093/ehjopen/oeae062)
Supplement: oeae062_Supplementary_Data [file oeae062_supplementary_data.zip › supplemental_figs_2024-06-01.docx]

Colchicine for cardiovascular and limb risk reduction in Medicare beneficiaries with peripheral artery disease: emulation of target trials

Patrick Heindel, MD, MPH; James J. Fitzgibbon, MD; Eric Secemsky, MD, MSc;

Deepak L. Bhatt, MD, MPH; Mohammed Al-Omran, MD, MSc; Subodh Verma, MD, PhD; Ibrahim A. Almaghlouth, MBBS, MSc; Arin Madenci, MD, PhD;

Mohamad A. Hussain, MD, PhD

Supplemental Material

**Supplemental Figure S1.** Covariate balance plot (Love plot) depicting imbalances at baseline between those treated with colchicine vs. non-steroidal anti-inflammatories at the time of urate lowering therapy initiation. Unadjusted estimates (red) and weighted estimates (blue) of the absolute mean differences (standardized for continuous variables) are presented. The dotted line represents the 10% threshold for residual imbalances after inverse probability weighting. CKD: chronic kidney disease; COPD: chronic obstructive pulmonary disease; MALE: major adverse limb event (above-ankle amputation, bypass graft, stenting, graft revision, thrombolysis, thrombectomy); MACE: major adverse cardiovascular event (acute myocardial infarction, stroke, coronary revascularization)


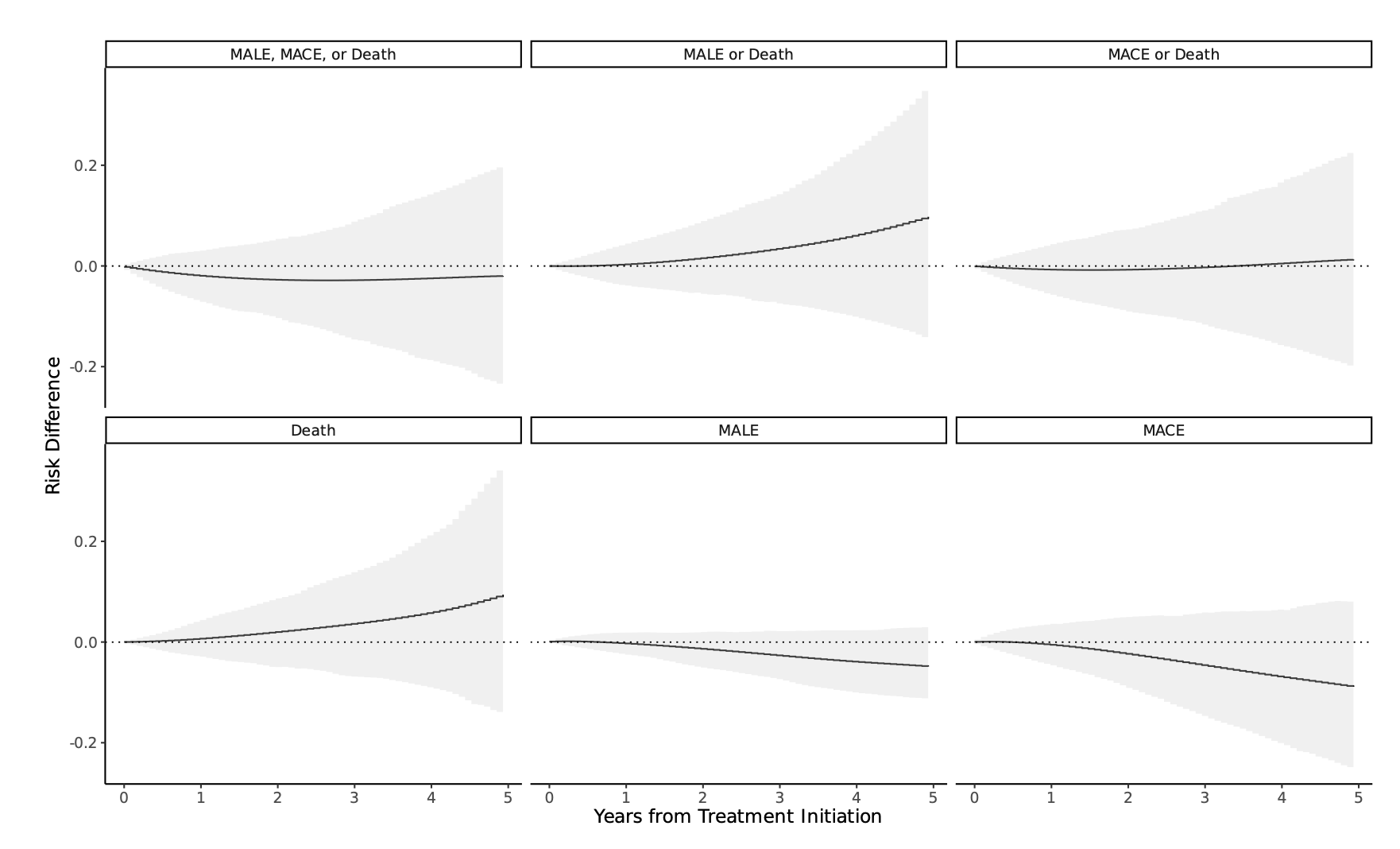


**Supplemental Figure S2.** Risk differences for the primary and secondary outcomes in Trial 2 from treatment initiation to 5 years. Shaded area represents percentile-based 95% confidence intervals generated with bootstrapping. MALE: major adverse limb event (above-ankle amputation, bypass graft, stenting, graft revision, thrombolysis, thrombectomy); MACE: major adverse cardiovascular event (acute myocardial infarction, stroke, coronary revascularization)


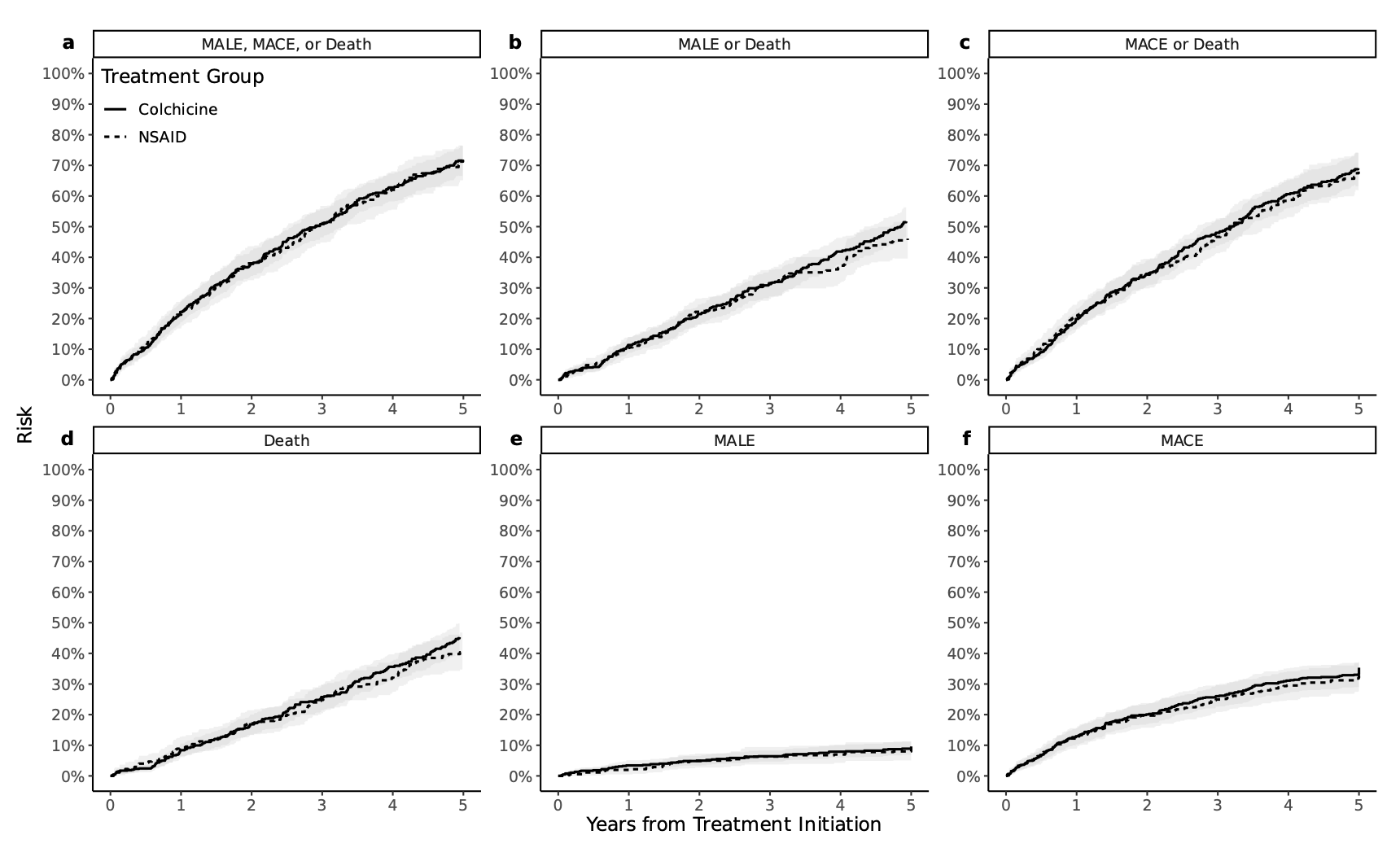


**Supplemental** **Figure S3.** Subgroup analysis of patients with coronary artery disease at the time of enrollment. Risk of the primary and secondary outcomes in Trial 1 from treatment initiation to 5 years. Solid line represents the treatment group (colchicine), dotted line represents control (NSAID). Shaded area represents percentile-based 95% confidence intervals generated with bootstrapping. MALE: major adverse limb event (above-ankle amputation, bypass graft, stenting, graft revision, thrombolysis, thrombectomy); MACE: major adverse cardiovascular event (acute myocardial infarction, stroke, coronary revascularization); NSAID: non-steroidal anti-inflammatory drug.


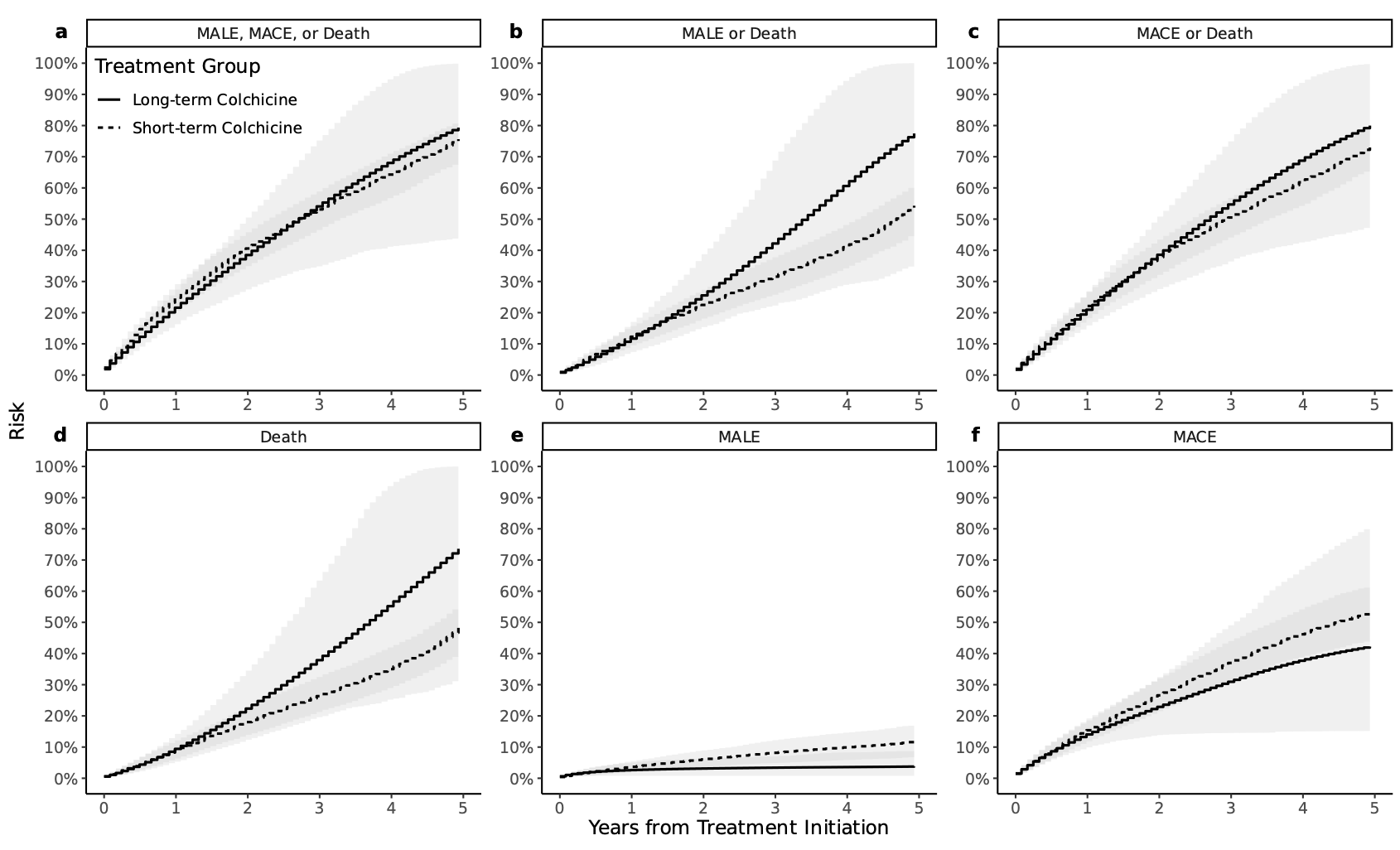


**Supplemental Figure S4.** Subgroup analysis of patients with coronary artery disease at the time of enrollment. Risk of the primary and secondary outcomes in Trial 2 from treatment initiation to 5 years. Solid line represents the treatment group (long-term colchicine), dotted line represents control (short-term colchicine). Shaded area represents percentile-based 95% confidence intervals generated with bootstrapping. MALE: major adverse limb event (above-ankle amputation, bypass graft, stenting, graft revision, thrombolysis, thrombectomy); MACE: major adverse cardiovascular event (acute myocardial infarction, stroke, coronary revascularization); NSAID: non-steroidal anti-inflammatory drug.


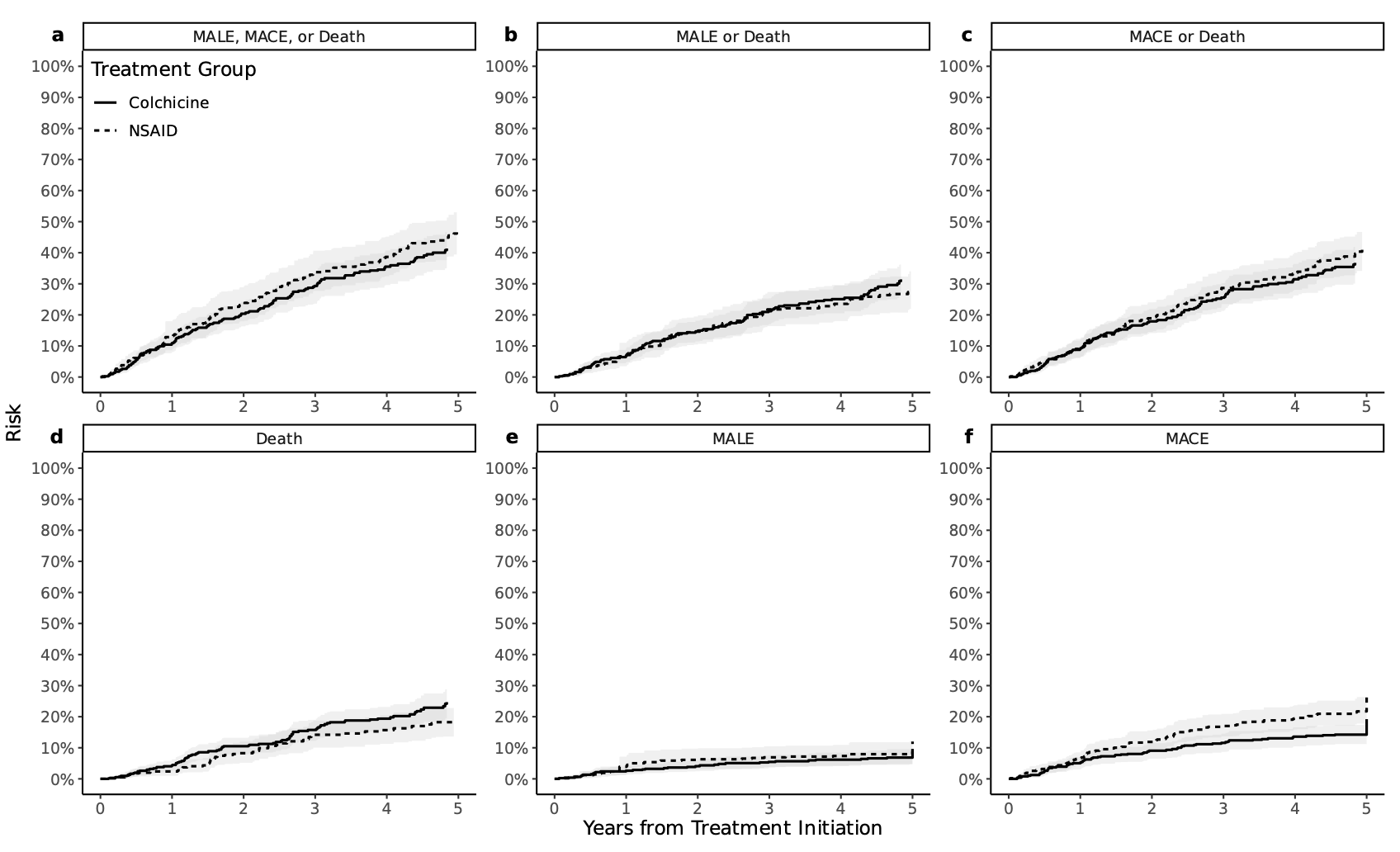


**Supplemental** **Figure S5.** Subgroup analysis of patients without coronary artery disease at the time of enrollment. Risk of the primary and secondary outcomes in Trial 1 from treatment initiation to 5 years. Solid line represents the treatment group (colchicine), dotted line represents control (NSAID). Shaded area represents percentile-based 95% confidence intervals generated with bootstrapping. MALE: major adverse limb event (above-ankle amputation, bypass graft, stenting, graft revision, thrombolysis, thrombectomy); MACE: major adverse cardiovascular event (acute myocardial infarction, stroke, coronary revascularization); NSAID: non-steroidal anti-inflammatory drug.


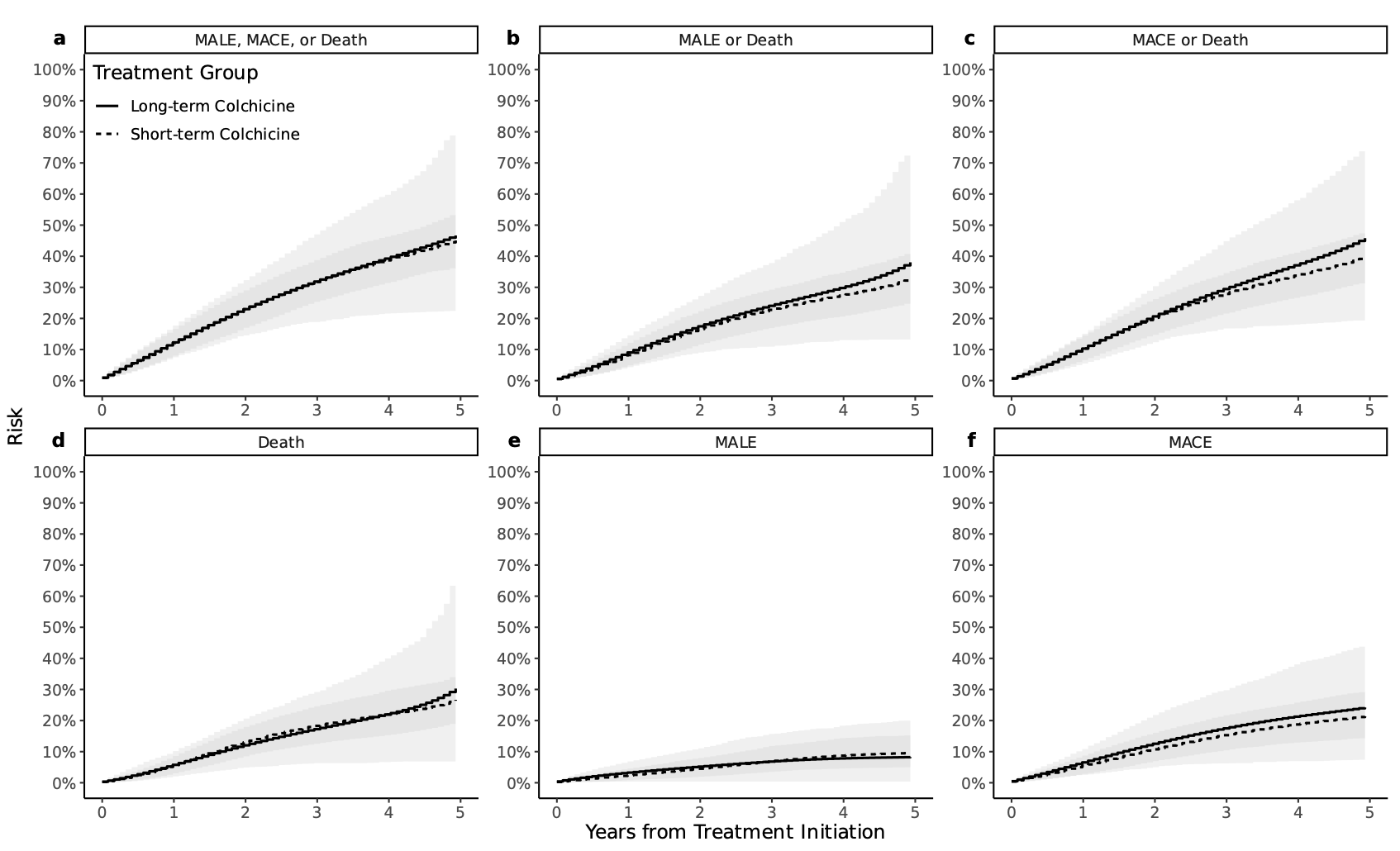


**Supplemental Figure S6.** Subgroup analysis of patients without coronary artery disease at the time of enrollment. Risk of the primary and secondary outcomes in Trial 2 from treatment initiation to 5 years. Solid line represents the treatment group (long-term colchicine), dotted line represents control (short-term colchicine). Shaded area represents percentile-based 95% confidence intervals generated with bootstrapping. MALE: major adverse limb event (above-ankle amputation, bypass graft, stenting, graft revision, thrombolysis, thrombectomy); MACE: major adverse cardiovascular event (acute myocardial infarction, stroke, coronary revascularization); NSAID: non-steroidal anti-inflammatory drug.
