# Supplementary material for: Colchicine for cardiovascular and limb risk reduction in Medicare beneficiaries with peripheral artery disease: emulation of target trials
Source: Eur Heart J Open. 2024 Aug 13;4(4):oeae062. doi: 10.1093/ehjopen/oeae062 (PMC11339712; doi:10.1093/ehjopen/oeae062)
Supplement: oeae062_Supplementary_Data [file oeae062_supplementary_data.zip › supplemental_tbls_2024-06-01.docx]

Colchicine for cardiovascular and limb risk reduction in Medicare beneficiaries with peripheral artery disease: emulation of target trials

Patrick Heindel, MD, MPH; James J. Fitzgibbon, MD; Eric Secemsky, MD, MSc;

Deepak L. Bhatt, MD, MPH; Mohammed Al-Omran, MD, MSc; Subodh Verma, MD, PhD; Ibrahim A. Almaghlouth, MBBS, MSc; Arin Madenci, MD, PhD;

Mohamad A. Hussain, MD, PhD

Supplemental Material

| **Protocol**  **Component** | **Target Trial Specification**  **(Hypothetical Randomized Trial)** | **Target Trial Emulation**  **(Using Observational MGB-Medicare Data)** |
| --- | --- | --- |
| **Eligibility Criteria** | - Patients receiving care at MGB between July 1, 2007, and December 31, 2019 - Medicare beneficiary enrolled in parts A, B, and D for ≥6 months - Age ≥66 - Diagnoses of PAD and gout - Initiation of urate lowering therapy - No prior colchicine, NSAID, or urate lowering therapy use - No allergy to colchicine or NSAIDs - No advanced CKD (≥ Stage 4) or hepatic failure - No prior enrollment in the present trial | Same as for the target trial, except:   - Medical histories obtained through ICD diagnosis codes - Diagnosis of PAD and gout based on primary ICD diagnosis codes for clinical encounters - PAD additionally defined as any prior revascularization or amputation for PAD, or ankle-brachial index <0.9 recorded in MGB electronic health record - Medication use determined from Medicare prescription dispensing claims - Allergies determined from MGB electronic health record |
| **Treatment Strategies** | - Colchicine (≥0.5mg daily) initiation (treatment) - NSAID initiation (control) | The date of medication initiation was defined as the first date of a prescription |
| **Treatment Assignment** | Participants are randomly assigned to a treatment strategy without blinding at the time of urate lowering therapy initiation | Emulation of randomization via inverse probability treatment weighting at baseline using potential confounders specified *a priori* |
| **Outcomes** | - Primary: 2-year composite of MALE, MACE, or all-cause mortality - Secondary: Individual components of composite outcome | Same as for the target trial. Outcomes were ascertained with Medicare claims codes |
| **Follow-up** | - Follow-up starts on the date of initiation of urate lowering therapy (baseline) - Follow-up ends at plan disenrollment, outcome, administrative censoring after 5 years, or at conclusion of the trial on December 31, 2019, whichever occurs first | Same as for the target trial |
| **Causal Contrasts** | Intention-to-treat effect: The effect of being assigned to colchicine or NSAID when initiating urate lowering therapy | Observational analog of the intention-to-treat effect |
| **Statistical Analysis** | - Compare cumulative incidence (risk) curves, risk differences, and risk ratios for all outcomes - Subgroup analysis of those with comorbid coronary artery disease at baseline - Total effects estimated for secondary outcomes (*i.e.*, MALE, MACE) subject to competing event of death | Same as for the target trial |
| MGB: Mass General Brigham Health System; PAD: peripheral artery disease; NSAID: non-steroidal anti-inflammatory drug; CKD: chronic kidney disease; ICD: International Classification of Diseases; MACE: admission for myocardial infarction, acute stroke, or coronary revascularization; MALE: above-ankle amputation, iliac arterial revascularization, or infrainguinal arterial revascularization | | |

**Supplemental Table S1.** Target trial protocol with corresponding emulation methodology for Trial 1.

| **Protocol**  **Component** | **Target Trial Specification**  **(Hypothetical Randomized Trial)** | **Target Trial Emulation**  **(Using Observational MGB-Medicare Data)** |
| --- | --- | --- |
| **Eligibility Criteria** | - Patients receiving care at MGB between July 1, 2007, and December 31, 2019 - Medicare beneficiary enrolled in parts A, B, and D for ≥6 months - Age ≥66 at time of enrollment - Diagnoses of PAD and gout - Initiation of urate lowering therapy - No prior colchicine, NSAID, or urate lowering therapy use - No allergy to colchicine or NSAIDs - No advanced CKD (≥ Stage 4) or hepatic failure - No prior enrollment in the present trial | Same as for the target trial, except:   - Medical histories obtained through ICD diagnosis codes - Diagnosis of PAD and gout based on primary ICD diagnosis codes for clinical encounters - PAD additionally defined as any prior revascularization or amputation for PAD, or ankle-brachial index <0.9 recorded in MGB electronic health record - Medication use determined from Medicare prescription dispensing claims - Allergies determined from MGB electronic health record |
| **Treatment Strategies** | - Long-term colchicine therapy (treatment), where ≥0.5mg of colchicine must be taken daily until the end of the trial. - Short-term colchicine therapy (control), where ≥0.5mg of colchicine must be taken daily for 90 days. Colchicine use after 90 days is considered a protocol deviation. | Same as for the target trial. Medication use is ascertained through prescription dispensing claims data and the date of medication initiation was defined as the first date of a prescription. |
| **Treatment Assignment** | Participants are randomly assigned to a treatment strategy without blinding at the time of urate lowering therapy initiation | Clones are assigned to each treatment strategy at baseline |
| **Outcomes** | - Primary: 2-year composite of MALE, MACE, or all-cause mortality - Secondary: Individual components of composite outcome with acknowledgement of competing events | Same as for the target trial. Outcomes were ascertained with Medicare claims codes. |
| **Follow-up** | - Follow-up starts on the date of concurrent initiation of urate lowering therapy and either of the treatment strategies (baseline) - Follow-up ends at plan disenrollment, outcome, administrative censoring after 5 years, or at conclusion of the trial on December 31, 2019, whichever occurs first | Same as for the target trial |
| **Causal Contrasts** | - Intention-to-treat effect - Per-protocol effect | Observational analog of the per-protocol effect |
| **Statistical Analysis** | - Intention-to-treat analysis - Non-naïve per-protocol analysis with inverse probability weighting to avoid selection bias (*i.e.*, collider-stratification bias) introduced by censoring at the time of protocol deviation - Compare cumulative incidence (risk) curves, risk differences, and risk ratios for all outcomes - Subgroup analysis of those with comorbid coronary artery disease at baseline - Total effects estimated for secondary outcomes (i.e., MALE, MACE) subject to competing event of death | Same as the per-protocol analysis of the target trial |
| MGB: Mass General Brigham Health System; PAD: peripheral artery disease; NSAID: non-steroidal anti-inflammatory drug; CKD: chronic kidney disease; ICD: International Classification of Diseases; MACE: admission for myocardial infarction, acute stroke, or coronary revascularization; MALE: above-ankle amputation, iliac arterial revascularization, or infrainguinal arterial revascularization | | |

**Supplemental Table S2.** Target trial protocol with corresponding emulation methodology for Trial 2.

| **Covariate** | **Codes** |
| --- | --- |
| Advanced CKD (Stage ≥4) | ICD9:5854, ICD9:5855, ICD9:5856, ICD10:I120, ICD10:I1311, ICD10:I132, ICD10:N184, ICD10:N185, ICD10:N186 |
| CKD (Stage 1-3) | ICD10:I120, ICD10:I129, ICD10:I1310, ICD10:I1311, ICD10:I132, ICD10:N181, ICD10:N182, ICD10:N183, ICD10:N184, ICD10:N185, ICD10:N186, ICD10:N189 |
| COPD | ICD9:490, ICD9:4910, ICD9:4911, ICD9:49120, ICD9:49121, ICD9:49122, ICD9:4918, ICD9:4919, ICD9:4920, ICD9:4928, ICD9:4940, ICD9:496, ICD10:J410, ICD10:J411, ICD10:J418, ICD10:J42, ICD10:J430, ICD10:J431, ICD10:J432, ICD10:J438, ICD10:J439, ICD10:J440, ICD10:J441, ICD10:J449, ICD10:J470, ICD10:J471, ICD10:J479 |
| Cerebrovascular Disease | ICD9:430, ICD9:431, ICD9:4320, ICD9:4321, ICD9:4329, ICD9:43311, ICD9:43321, ICD9:43381, ICD9:43400, ICD9:43401, ICD9:43410, ICD9:43411, ICD9:43490, ICD9:43491, ICD9:4350, ICD9:4352, ICD9:4353, ICD9:4358, ICD9:4359, ICD9:436, ICD9:4380, ICD9:43811, ICD9:43812, ICD9:43820, ICD9:43821, ICD9:43822, ICD9:43830, ICD9:4387, ICD9:43882, ICD9:43883, ICD9:43884, ICD9:43885, ICD9:43889, ICD9:4389, ICD10:I609, ICD10:I610, ICD10:I618, ICD10:I619, ICD10:I6200, ICD10:I6201, ICD10:I6202, ICD10:I621, ICD10:I629, ICD10:I6502, ICD10:I6503, ICD10:I6521, ICD10:I6522, ICD10:I6523, ICD10:I6529, ICD10:I658, ICD10:I6622, ICD10:I669, ICD10:I69021, ICD10:I69051, ICD10:I69122, ICD10:I69191, ICD10:I6930, ICD10:I69311, ICD10:I69312, ICD10:I69315, ICD10:I69318, ICD10:I69319, ICD10:I69320, ICD10:I69322, ICD10:I69328, ICD10:I69331, ICD10:I69349, ICD10:I69351, ICD10:I69352, ICD10:I69354, ICD10:I69359, ICD10:I69390, ICD10:I69391, ICD10:I69392, ICD10:I69393, ICD10:I69398, ICD10:I6980, ICD10:I69851, ICD10:I69854, ICD10:I69898, ICD10:I6990, ICD10:I69920, ICD10:I69951, ICD10:I69959, ICD10:I69965, ICD10:I69992, ICD10:I69998 |
| Coronary Artery Disease | ICD9:4110, ICD9:4111, ICD9:41181, ICD9:41189, ICD9:412, ICD9:4130, ICD9:4139, ICD9:41400, ICD9:41401, ICD9:41406, ICD9:4142, ICD9:4143, ICD9:4144, ICD9:4148, ICD9:4149, ICD9:V4581, ICD9:V4582, ICD10:I200, ICD10:I208, ICD10:I209, ICD10:I240, ICD10:I248, ICD10:I249, ICD10:I2510, ICD10:I25110, ICD10:I25118, ICD10:I25119, ICD10:I252, ICD10:I255, ICD10:I25700, ICD10:I25701, ICD10:I25708, ICD10:I25709, ICD10:I25718, ICD10:I25719, ICD10:I25739, ICD10:I25758, ICD10:I25810, ICD10:I2589, ICD10:I259 |
| Diabetes | ICD9:24900, ICD9:24901, ICD9:24911, ICD9:24940, ICD9:24960, ICD9:24991, ICD9:25000, ICD9:25001, ICD9:25002, ICD9:25003, ICD9:25012, ICD9:25020, ICD9:25022, ICD9:25030, ICD9:25040, ICD9:25041, ICD9:25042, ICD9:25043, ICD9:25050, ICD9:25051, ICD9:25052, ICD9:25053, ICD9:25060, ICD9:25061, ICD9:25062, ICD9:25063, ICD9:25070, ICD9:25071, ICD9:25072, ICD9:25073, ICD9:25080, ICD9:25081, ICD9:25082, ICD9:25083, ICD9:25090, ICD9:25091, ICD9:25092, ICD9:25093, ICD9:79021, ICD9:79022, ICD9:79029, ICD10:E1021, ICD10:E1022, ICD10:E1029, ICD10:E10319, ICD10:E10321, ICD10:E103211, ICD10:E103213, ICD10:E10329, ICD10:E103291, ICD10:E103292, ICD10:E103293, ICD10:E10339, ICD10:E103512, ICD10:E10359, ICD10:E1039, ICD10:E1040, ICD10:E1042, ICD10:E1051, ICD10:E1059, ICD10:E10610, ICD10:E10622, ICD10:E10628, ICD10:E1065, ICD10:E108, ICD10:E109, ICD10:E1100, ICD10:E1101, ICD10:E1110, ICD10:E1121, ICD10:E1122, ICD10:E1129, ICD10:E11311, ICD10:E11319, ICD10:E11321, ICD10:E113211, ICD10:E113213, ICD10:E113219, ICD10:E11329, ICD10:E113292, ICD10:E113293, ICD10:E113299, ICD10:E113311, ICD10:E113312, ICD10:E113313, ICD10:E11339, ICD10:E113392, ICD10:E113393, ICD10:E113412, ICD10:E113413, ICD10:E11349, ICD10:E113511, ICD10:E113512, ICD10:E113513, ICD10:E113519, ICD10:E113551, ICD10:E113552, ICD10:E11359, ICD10:E113591, ICD10:E113593, ICD10:E113599, ICD10:E1136, ICD10:E1139, ICD10:E1140, ICD10:E1141, ICD10:E1142, ICD10:E1143, ICD10:E1144, ICD10:E1149, ICD10:E1151, ICD10:E1152, ICD10:E1159, ICD10:E11610, ICD10:E11618, ICD10:E11620, ICD10:E11621, ICD10:E11622, ICD10:E11628, ICD10:E11649, ICD10:E1165, ICD10:E1169, ICD10:E118, ICD10:E119, ICD10:E1310, ICD10:E1321, ICD10:E1322, ICD10:E1329, ICD10:E13359, ICD10:E1342, ICD10:E1349, ICD10:E13621, ICD10:E13628, ICD10:E1365, ICD10:E1369, ICD10:E138, ICD10:E139 |
| Gout | ICD9:2740, ICD9:27400, ICD9:27401, ICD9:27402, ICD9:27403, ICD9:27410, ICD9:27411, ICD9:27481, ICD9:27482, ICD9:27489, ICD9:2749, ICD9:71210, ICD9:71220, ICD9:71224, ICD9:71226, ICD9:71227, ICD9:71230, ICD9:71233, ICD9:71234, ICD9:71236, ICD9:71237, ICD9:71238, ICD9:71239, ICD9:71293, ICD9:71296, ICD9:71299, ICD10:M1000, ICD10:M10011, ICD10:M10019, ICD10:M10021, ICD10:M10031, ICD10:M10032, ICD10:M10041, ICD10:M10042, ICD10:M10049, ICD10:M10051, ICD10:M10061, ICD10:M10062, ICD10:M10069, ICD10:M10071, ICD10:M10072, ICD10:M10079, ICD10:M1008, ICD10:M1009, ICD10:M1030, ICD10:M10331, ICD10:M10341, ICD10:M10349, ICD10:M10361, ICD10:M10362, ICD10:M10371, ICD10:M10372, ICD10:M10379, ICD10:M1039, ICD10:M10461, ICD10:M10462, ICD10:M10471, ICD10:M10472, ICD10:M109, ICD10:M1A00X0, ICD10:M1A00X1, ICD10:M1A0211, ICD10:M1A0220, ICD10:M1A0310, ICD10:M1A0320, ICD10:M1A0410, ICD10:M1A0411, ICD10:M1A0421, ICD10:M1A0490, ICD10:M1A0491, ICD10:M1A0610, ICD10:M1A0620, ICD10:M1A0621, ICD10:M1A0690, ICD10:M1A0710, ICD10:M1A0711, ICD10:M1A0720, ICD10:M1A0721, ICD10:M1A0790, ICD10:M1A0791, ICD10:M1A09X0, ICD10:M1A09X1, ICD10:M1A1510, ICD10:M1A30X0, ICD10:M1A30X1, ICD10:M1A3410, ICD10:M1A3411, ICD10:M1A3421, ICD10:M1A3610, ICD10:M1A3710, ICD10:M1A3711, ICD10:M1A3720, ICD10:M1A3721, ICD10:M1A3790, ICD10:M1A3791, ICD10:M1A39X0, ICD10:M1A39X1, ICD10:M1A4310, ICD10:M1A4321, ICD10:M1A4410, ICD10:M1A4710, ICD10:M1A49X0, ICD10:M1A9XX0, ICD10:M1A9XX1 |
| Heart Failure | ICD9:39891, ICD9:4280, ICD9:4281, ICD9:42820, ICD9:42821, ICD9:42822, ICD9:42823, ICD9:42830, ICD9:42831, ICD9:42832, ICD9:42833, ICD9:42840, ICD9:42841, ICD9:42842, ICD9:42843, ICD9:4289, ICD10:I110, ICD10:I130, ICD10:I501, ICD10:I5020, ICD10:I5021, ICD10:I5022, ICD10:I5023, ICD10:I5030, ICD10:I5031, ICD10:I5032, ICD10:I5033, ICD10:I5040, ICD10:I5041, ICD10:I5042, ICD10:I5043, ICD10:I50810, ICD10:I50811, ICD10:I50812, ICD10:I50813, ICD10:I5082, ICD10:I5084, ICD10:I5089, ICD10:I509 |
| Hepatic Failure | ICD9:5712, ICD9:5715, ICD10:K7200, ICD10:K7290, ICD10:K7291 |
| Hypertension | ICD9:4010, ICD9:4011, ICD9:4019, ICD9:40200, ICD9:40210, ICD9:40211, ICD9:40290, ICD9:40291, ICD9:40300, ICD9:40301, ICD9:40310, ICD9:40311, ICD9:40390, ICD9:40391, ICD9:40400, ICD9:40401, ICD9:40410, ICD9:40411, ICD9:40413, ICD9:40490, ICD9:40491, ICD9:40493, ICD9:40501, ICD9:40511, ICD9:40591, ICD9:4372, ICD10:I10, ICD10:I119, ICD10:I150, ICD10:I151, ICD10:I152, ICD10:I158, ICD10:I159, ICD10:I160, ICD10:I161, ICD10:I674 |
| MACE  MACE, continued | ICD9-PCS:3604, ICD9-PCS:3606, ICD9-PCS:3607, ICD9-PCS:3609, ICD9-PCS:3610, ICD9-PCS:3611, ICD9-PCS:3612, ICD9-PCS:3613, ICD9-PCS:3614, ICD9-PCS:3615, ICD9-PCS:3616, ICD9-PCS:3617, ICD9-PCS:3619, ICD9-PCS:362, ICD9-PCS:3631, ICD9-PCS:3632, ICD9-PCS:3633, ICD9-PCS:3634, ICD9-PCS:3639, ICD9:41000, ICD9:41001, ICD9:41002, ICD9:41010, ICD9:41011, ICD9:41012, ICD9:41020, ICD9:41021, ICD9:41022, ICD9:41030, ICD9:41031, ICD9:41032, ICD9:41040, ICD9:41041, ICD9:41042, ICD9:41050, ICD9:41051, ICD9:41052, ICD9:41060, ICD9:41061, ICD9:41062, ICD9:41070, ICD9:41071, ICD9:41072, ICD9:41080, ICD9:41081, ICD9:41082, ICD9:41090, ICD9:41091, ICD9:41092, ICD9:430, ICD9:431, ICD9:4320, ICD9:4321, ICD9:4329, ICD9:43300, ICD9:43301, ICD9:43310, ICD9:43311, ICD9:43320, ICD9:43321, ICD9:43330, ICD9:43331, ICD9:43380, ICD9:43381, ICD9:43390, ICD9:43391, ICD9:43400, ICD9:43401, ICD9:43410, ICD9:43411, ICD9:43490, ICD9:43491, ICD9:4350, ICD9:4351, ICD9:4352, ICD9:4353, ICD9:4358, ICD9:4359, ICD9:436, ICD10-PCS:0210, ICD10-PCS:0211, ICD10-PCS:0212, ICD10-PCS:0213, ICD10-PCS:0270, ICD10-PCS:0271, ICD10-PCS:0272, ICD10-PCS:0273, ICD10:G459, ICD10:I2101, ICD10:I2102, ICD10:I2109, ICD10:I2111, ICD10:I2119, ICD10:I2121, ICD10:I2129, ICD10:I213, ICD10:I214, ICD10:I219, ICD10:I21A1, ICD10:I21A9, ICD10:I220, ICD10:I221, ICD10:I222, ICD10:I228, ICD10:I229, ICD10:I6300, ICD10:I63011, ICD10:I63012, ICD10:I63013, ICD10:I63019, ICD10:I6302, ICD10:I63031, ICD10:I63032, ICD10:I63033, ICD10:I63039, ICD10:I6309, ICD10:I6310, ICD10:I63111, ICD10:I63112, ICD10:I63113, ICD10:I63119, ICD10:I6312, ICD10:I63131, ICD10:I63132, ICD10:I63133, ICD10:I63139, ICD10:I6319, ICD10:I6320, ICD10:I63211, ICD10:I63212, ICD10:I63213, ICD10:I63219, ICD10:I6322, ICD10:I63231, ICD10:I63232, ICD10:I63233, ICD10:I63239, ICD10:I6329, ICD10:I6330, ICD10:I63311, ICD10:I63312, ICD10:I63313, ICD10:I63319, ICD10:I63321, ICD10:I63322, ICD10:I63323, ICD10:I63329, ICD10:I63331, ICD10:I63332, ICD10:I63333, ICD10:I63339, ICD10:I63341, ICD10:I63342, ICD10:I63343, ICD10:I63349, ICD10:I6339, ICD10:I6340, ICD10:I63411, ICD10:I63412, ICD10:I63413, ICD10:I63419, ICD10:I63421, ICD10:I63422, ICD10:I63423, ICD10:I63429, ICD10:I63431, ICD10:I63432, ICD10:I63433, ICD10:I63439, ICD10:I63441, ICD10:I63442, ICD10:I63443, ICD10:I63449, ICD10:I6349, ICD10:I6350, ICD10:I63511, ICD10:I63512, ICD10:I63513, ICD10:I63519, ICD10:I63521, ICD10:I63522, ICD10:I63523, ICD10:I63529, ICD10:I63531, ICD10:I63532, ICD10:I63533, ICD10:I63539, ICD10:I63541, ICD10:I63542, ICD10:I63543, ICD10:I63549, ICD10:I6359, ICD10:I636, ICD10:I6381, ICD10:I6389, ICD10:I639, CPT4:33510, CPT4:33511, CPT4:33512, CPT4:33513, CPT4:33517, CPT4:33518, CPT4:33519, CPT4:33521, CPT4:33522, CPT4:33530, CPT4:33533, CPT4:33534, CPT4:33535, CPT4:33536, CPT4:33572, CPT4:33863, CPT4:33864, CPT4:92920, CPT4:92921, CPT4:92924, CPT4:92928, CPT4:92929, CPT4:92933, CPT4:92937, CPT4:92941, CPT4:92943, CPT4:92944, CPT4:92973, CPT4:92974, CPT4:92977, CPT4:92980, CPT4:92981, CPT4:92982, CPT4:92984, CPT4:92985, CPT4:92995, CPT4:92996 |
| MALE | ICD9-PCS:0055, ICD9-PCS:0060, ICD9-PCS:3818, ICD9-PCS:3838, ICD9-PCS:3848, ICD9-PCS:3925, ICD9-PCS:3929, ICD9-PCS:3950, ICD9-PCS:3990, ICD9-PCS:8410, ICD9-PCS:8413, ICD9-PCS:8414, ICD9-PCS:8415, ICD9-PCS:8416, ICD9-PCS:8417, ICD9-PCS:8418, ICD10-PCS:041C, ICD10-PCS:041D, ICD10-PCS:041E, ICD10-PCS:041F, ICD10-PCS:041H, ICD10-PCS:041J, ICD10-PCS:041K, ICD10-PCS:041L, ICD10-PCS:041M, ICD10-PCS:041N, ICD10-PCS:041P, ICD10-PCS:041Q, ICD10-PCS:041R, ICD10-PCS:041S, ICD10-PCS:041T, ICD10-PCS:041U, ICD10-PCS:047C, ICD10-PCS:047D, ICD10-PCS:047E, ICD10-PCS:047F, ICD10-PCS:047H, ICD10-PCS:047J, ICD10-PCS:047K, ICD10-PCS:047L, ICD10-PCS:047M, ICD10-PCS:047N, ICD10-PCS:047P, ICD10-PCS:047Q, ICD10-PCS:047R, ICD10-PCS:047S, ICD10-PCS:047T, ICD10-PCS:047U, ICD10-PCS:04HC, ICD10-PCS:04HD, ICD10-PCS:04HE, ICD10-PCS:04HF, ICD10-PCS:04HH, ICD10-PCS:04HJ, ICD10-PCS:04HK, ICD10-PCS:04HL, ICD10-PCS:04HM, ICD10-PCS:04HN, ICD10-PCS:04HP, ICD10-PCS:04HQ, ICD10-PCS:04HR, ICD10-PCS:04HS, ICD10-PCS:04HT, ICD10-PCS:04HU, ICD10-PCS:04RC, ICD10-PCS:04RD, ICD10-PCS:04RE, ICD10-PCS:04RF, ICD10-PCS:04RH, ICD10-PCS:04RJ, ICD10-PCS:04RK, ICD10-PCS:04RL, ICD10-PCS:04RM, ICD10-PCS:04RN, ICD10-PCS:04RP, ICD10-PCS:04RQ, ICD10-PCS:04RR, ICD10-PCS:04RS, ICD10-PCS:04RT, ICD10-PCS:04RU, ICD10-PCS:0Y62, ICD10-PCS:0Y63, ICD10-PCS:0Y64, ICD10-PCS:0Y67, ICD10-PCS:0Y68, ICD10-PCS:0Y6C, ICD10-PCS:0Y6D, ICD10-PCS:0Y6F, ICD10-PCS:0Y6G, ICD10-PCS:0Y6H, ICD10-PCS:0Y6J, CPT4:27590, CPT4:27592, CPT4:27596, CPT4:27598, CPT4:27880, CPT4:27882, CPT4:27886, CPT4:27889, CPT4:34201, CPT4:34203, CPT4:35351, CPT4:35355, CPT4:35361, CPT4:35363, CPT4:35371, CPT4:35372, CPT4:35381, CPT4:35454, CPT4:35456, CPT4:35459, CPT4:35470, CPT4:35473, CPT4:35474, CPT4:35482, CPT4:35483, CPT4:35485, CPT4:35492, CPT4:35493, CPT4:35495, CPT4:35521, CPT4:35533, CPT4:35537, CPT4:35538, CPT4:35539, CPT4:35540, CPT4:35541, CPT4:35546, CPT4:35548, CPT4:35549, CPT4:35551, CPT4:35556, CPT4:35558, CPT4:35563, CPT4:35565, CPT4:35566, CPT4:35571, CPT4:35582, CPT4:35583, CPT4:35585, CPT4:35587, CPT4:35623, CPT4:35637, CPT4:35638, CPT4:35641, CPT4:35646, CPT4:35647, CPT4:35651, CPT4:35654, CPT4:35656, CPT4:35661, CPT4:35663, CPT4:35665, CPT4:35666, CPT4:35671, CPT4:35879, CPT4:35881, CPT4:35883, CPT4:35884, CPT4:37184, CPT4:37185, CPT4:37186, CPT4:37205, CPT4:37206, CPT4:37207, CPT4:37208, CPT4:37220, CPT4:37221, CPT4:37222, CPT4:37223, CPT4:37224, CPT4:37225, CPT4:37226, CPT4:37227, CPT4:37228, CPT4:37229, CPT4:37230, CPT4:37231, CPT4:37232, CPT4:37233, CPT4:37234, CPT4:37235 |
| Peripheral Artery Disease  Peripheral Artery Disease, continued | ICD9:4400, ICD9:4401, ICD9:44020, ICD9:44021, ICD9:44022, ICD9:44023, ICD9:44029, ICD9:4408, ICD9:4409, ICD9:4439, ICD9:5570, ICD9:5571, ICD9:5579, ICD10:I700, ICD10:I701, ICD10:I70201, ICD10:I70202, ICD10:I70203, ICD10:I70208, ICD10:I70209, ICD10:I70211, ICD10:I70212, ICD10:I70213, ICD10:I70219, ICD10:I70221, ICD10:I70222, ICD10:I70223, ICD10:I70229, ICD10:I70232, ICD10:I70233, ICD10:I70234, ICD10:I70235, ICD10:I70239, ICD10:I70242, ICD10:I70243, ICD10:I70244, ICD10:I70245, ICD10:I70249, ICD10:I7025, ICD10:I70261, ICD10:I70262, ICD10:I70291, ICD10:I70293, ICD10:I70299, ICD10:I70302, ICD10:I70335, ICD10:I70345, ICD10:I70412, ICD10:I70421, ICD10:I70501, ICD10:I70513, ICD10:I708, ICD10:I7090, ICD10:I7091, ICD10:I7300, ICD10:I731, ICD10:I7381, ICD10:I7389, ICD10:I739 |
| Rest Pain | ICD9:44022, ICD10:I70221, ICD10:I70222, ICD10:I70223, ICD10:I70229, ICD10:I70421 |
| Tissue Loss | ICD9:44024, ICD9:70700, ICD9:70703, ICD9:70704, ICD9:70705, ICD9:70706, ICD9:70707, ICD9:70709, ICD9:70710, ICD9:70712, ICD9:70713, ICD9:70714, ICD9:70715, ICD9:70719, ICD9:70720, ICD9:70721, ICD9:70722, ICD9:70723, ICD9:70724, ICD9:70725, ICD9:7078, ICD9:7079, ICD9:7854, ICD9:88003, ICD9:88100, ICD9:88101, ICD9:8820, ICD9:8830, ICD9:8831, ICD9:8840, ICD9:8900, ICD9:8910, ICD9:8911, ICD9:8920, ICD9:8921, ICD9:8930, ICD9:8931, ICD9:8940, ICD9:8941, ICD9:8950, ICD9:8951, ICD9:8970, ICD9:8972, ICD9:8974, ICD9:9061, ICD10:I96, ICD10:L89101, ICD10:L89102, ICD10:L89109, ICD10:L89140, ICD10:L89143, ICD10:L89150, ICD10:L89151, ICD10:L89152, ICD10:L89153, ICD10:L89154, ICD10:L89159, ICD10:L89300, ICD10:L89301, ICD10:L89302, ICD10:L89304, ICD10:L89309, ICD10:L89310, ICD10:L89311, ICD10:L89312, ICD10:L89313, ICD10:L89314, ICD10:L89319, ICD10:L89320, ICD10:L89321, ICD10:L89322, ICD10:L89323, ICD10:L89324, ICD10:L89329, ICD10:L89520, ICD10:L89523, ICD10:L89524, ICD10:L89609, ICD10:L89610, ICD10:L89613, ICD10:L89620, ICD10:L89622, ICD10:L89623, ICD10:L89629, ICD10:L89890, ICD10:L89891, ICD10:L89892, ICD10:L89893, ICD10:L89894, ICD10:L89899, ICD10:L8990, ICD10:L8995, ICD10:L97129, ICD10:L97211, ICD10:L97212, ICD10:L97219, ICD10:L97221, ICD10:L97222, ICD10:L97302, ICD10:L97312, ICD10:L97319, ICD10:L97322, ICD10:L97323, ICD10:L97329, ICD10:L97411, ICD10:L97412, ICD10:L97419, ICD10:L97421, ICD10:L97422, ICD10:L97423, ICD10:L97429, ICD10:L97501, ICD10:L97509, ICD10:L97511, ICD10:L97512, ICD10:L97514, ICD10:L97516, ICD10:L97518, ICD10:L97519, ICD10:L97521, ICD10:L97522, ICD10:L97524, ICD10:L97529, ICD10:L97801, ICD10:L97811, ICD10:L97812, ICD10:L97819, ICD10:L97821, ICD10:L97822, ICD10:L97828, ICD10:L97829, ICD10:L97909, ICD10:L97911, ICD10:L97919, ICD10:L97921, ICD10:L97922, ICD10:L97929, ICD10:L98412, ICD10:L98419, ICD10:L98491, ICD10:L98492, ICD10:L98499, ICD10:S41011A, ICD10:S41109A, ICD10:S51011A, ICD10:S51012A, ICD10:S51802A, ICD10:S51802D, ICD10:S51812A, ICD10:S51812D, ICD10:S51822A, ICD10:S61002A, ICD10:S61011A, ICD10:S61012A, ICD10:S61228A, ICD10:S61401D, ICD10:S61409A, ICD10:S61411A, ICD10:S61412A, ICD10:S71102A, ICD10:S71109A, ICD10:S71111A, ICD10:S81001A, ICD10:S81001D, ICD10:S81002A, ICD10:S81011A, ICD10:S81011D, ICD10:S81012A, ICD10:S81801A, ICD10:S81801D, ICD10:S81802A, ICD10:S81802D, ICD10:S81809A, ICD10:S81809D, ICD10:S81811A, ICD10:S81812A, ICD10:S81812D, ICD10:S91001A, ICD10:S91002A, ICD10:S91002D, ICD10:S91009A, ICD10:S91009D, ICD10:S91011D, ICD10:S91101A, ICD10:S91101D, ICD10:S91102A, ICD10:S91104A, ICD10:S91104D, ICD10:S91105A, ICD10:S91109A, ICD10:S91109D, ICD10:S91112A, ICD10:S91114A, ICD10:S91114D, ICD10:S91115A, ICD10:S91202A, ICD10:S91209A, ICD10:S91301A, ICD10:S91301D, ICD10:S91302A, ICD10:S91302D, ICD10:S91309A |

**Supplemental Table S3.** Study variable definition details.
